# Photocatalytic Activity of Revolutionary *Galaxaura elongata, Turbinaria ornata,* and *Enteromorpha flexuosa*’s Bio-Capped Silver Nanoparticles for Industrial Wastewater Treatment

**DOI:** 10.3390/nano11123241

**Published:** 2021-11-29

**Authors:** Manal N. Abdel Azeem, Safwat Hassaballa, Osama M. Ahmed, Khaled N. M. Elsayed, Mohamed Shaban

**Affiliations:** 1 Physiology Division, Zoology Department, Faculty of Science, Beni-Suef University, Beni-Suef 62521, Egypt; sci.monia@yahoo.com (M.N.A.A.); osama.ahmed@science.bsu.edu.eg (O.M.A.); 2 Nanophotonics and Applications (NPA) Lab, Physics Department, Faculty of Science, Beni-Suef University, Beni-Suef 62521, Egypt; 3 Department of Physics, Faculty of Science, Islamic University in Madinah, AlMadinah Almonawara 42351, Saudi Arabia; safwat.hassaballa@iu.edu.sa; 4 Botany and Microbiology Department, Faculty of Science, Beni-Suef University, Beni-Suef 62521, Egypt; K.elsayed@science.bsu.edu.eg

**Keywords:** biogenic silver nanoparticles, macroalgae, photocatalysts, dye degradation, kinetics, mechanism

## Abstract

More suitable wastewater treatment schemes need to be developed to get rid of harmful dyes and pigments before they are discharged, primarily from apparel and textile factories, into water bodies. Silver nanoparticles (Ag-NPs) are very effective, reductive nanocatalysts that can degrade many organic dyes. In this study, Ag-NPs are stabilized and capped with bioactive compounds such as *Galaxaura elongata*, *Turbinaria ornata*, and *Enteromorpha flexuosa* from marine macroalgae extracts to produce Ag[GE], Ag[TE], and Ag[EE] NPs. The reduction of Ag ions and the production of Ag[GE], Ag[TE], and Ag[EE] NPs have been substantiated by UV–Vis spectroscopy, SEM, EDX, and XRD tests. The NPs are sphere and crystalline shaped in nature with dimensions ranging from 20 to 25 nm. The biosynthesized Ag[GE], Ag[TE], Ag[EE] NPs were applied to photodegrade hazardous pigments such as methylene blue, Congo red, safranine O, and crystal violet under sunlight irradiation. In addition to the stability analysis, various experimental parameters, including dye concentration, exposure period, photocatalyst dose, and temperature, were optimized to achieve 100% photodegradation of the dyes. Moreover, the thermodynamic and kinetic parameters were calculated and the impact of scavengers on the photocatalytic mechanism was also investigated.

## 1. Introduction

The procurement of pure water is now highly urgent for humanitarian aims and remains a great global challenge to the 21st century [1]. One of the most substantial water pollutants is chemicals resulting from industrial wastewater, such as pigments and dyes that have a dangerous impact on water resources. Moreover, the slightest concentration of these organic materials initiates significant impairment in marine ecosystems [2,3,4]. Additionally, these complexes alter the color of water. As a result, dye particles in water are a strong indicator of pollution. These pollutants are microscopic particles that obstruct sunlight from reaching marine plants and animals, as well as reducing photosynthetic particle emission in the environment [5,6,7]. The wastes generated during textile production, particularly during dying operation, have a significant impact on the environment, causing a change in the water’s natural qualities and altering the action of photosynthetic activities as well as the solubility of gases in water [8].

Dyes are organic compounds that are broadly used as coloring agents in industries, and they differ from each other in terms of chemical structure. Dyes are utilized for coloring textiles, which makes up approximately 60% of overall dye production. After completing the staining process, about 15% of dyes are wasted. The released dye complexes diffuse into water sources at concentrations ranging from 10 to 200 milligrams per liter, causing widespread water contamination [9,10,11]. The discharge of industrial effluents like fabrics, paper, printing, leather, cosmetics, plastics, pharmaceuticals, and food without dye removal has an impact on marine life [12,13]. Dyes are a trigger for human genetic alterations because they easily accumulate in living tissues [14]. Most dyes and their by-products are also toxic to both humans and animals [15,16]. Methylene blue is a hazardous substance that can cause eye discomfort, anemia, biliousness, and vomiting [17]. As a result, dealing with water that has been contaminated with methylene blue is a difficult operation. The annual release of methylene blue dye-polluted wastewater causes a plethora of ecological issues, including a high level of chemical oxygen demand (COD) that surpasses the deadly limit, endangering marine species [18].

In recent years, a variety of promising methods have been used to cleanse wastewater containing coloring dye particles or organic materials. Plasma-based methods utilizing AC-driven air plasma, microwave plasma jet, and arc plasma jet in contact with water have been applied to degrade organic compounds such as methylene blue and phenol [19,20,21]. Furthermore, other methods include ignition, biotreatment, ozonation, and adsorption using hard adsorbents [22]. Those approaches, however, highlight concerns such as (i) the generation of poisonous, volatile chemicals in the incineration process; (ii) the increase of foul odor in the biological purification process; and (iii) other parameters such as pH, salt ion concentration, and temperature in the ozonation technique. As a result of these factors, the photocatalysis method using metal ions offers a viable alternative to pigments and the elimination of dyes. This technologically advanced process has several advantages over other conventional procedures, and results in a set of safe substances [23]. Plasmonic nanostructures, such as Ag-NPs, have recently received a lot of attention because of their ability to photodegrade wastewater dyes. Concerning volume ratio, the special properties of nanoparticles may be assigned to the small particle dimension varying from 1 to 100 nm and a significant surface area, resulting in its extensive equipment in the fields of medicine, electronics, physics, electronics, chemistry, and molecular biology [24]. The chemical synthesis of plasmonic nanoparticles is often expensive, consumes dangerous chemicals, and undesirably affects diverse biological systems [25]. Biosynthesis of nanoparticles is currently the most used technique because; compared to chemical and physical techniques; it is natural, cost-saving, usable, eco-friendly, biocompatible, and nontoxic. Ag-NPs require special attention because of their enviable properties, including their high surface-to-volume ratios, as well as their photocatalytic, biosensing, and antimicrobial effects [26]. In particular, biosynthesis of Ag-NPs by biological agents such as algal extracts has gained significant interest in the area of nanotechnology in the last few decades [27]. Moreover, the green biosynthesis methods of Ag-NPs using seaweed extract are promising because they have potential bioactive compounds such as carbohydrates, proteins, steroids, alkaloids, phenols, and flavonoids that are used to minimize the oxidation state of the Ag^+^ ion from (I) to (0) [28,29].

Despite previous growing attention, the green biosynthesis of Ag-NPs still faces ongoing challenges, including low yields, time consumption, the availability of environmentally friendly solvents, the reducing/stabilizing of agents, and the controllability of the properties of Ag-NPs [30,31]. In this work, macroalgae (*Galaxaura elongata*, *Turbinaria ornata*, and *Enteromorpha flexuosa*) extracts are used and optimized for massive bioproduction of Ag-NPs. The developed Ag-NPs of the three macroalgae extracts were characterized using a UV/Vis spectrophotometer, SEM, EDX, and XRD. The application of the developed NPs was optimized for the decomposition of dissimilar hazardous dyes such as methylene blue, Congo red, safranin O, and crystal violet under sunlight irradiation in terms of dye concentration, exposure period, temperature, and catalyst dose. The thermodynamic and kinetic parameters are determined in addition to the discussion of the photocatalytic mechanism.

## 2. Materials and Methods

### 2.1. Materials

Silver nitrate (AgNO_3_) was procured from Sigma-Aldrich, (St. Louis, MO, USA). Different types of marine macroalgae, such as red macroalgae (*Galaxaura elongata*), brown macroalgae (*Turbinaria ornata*), and green macroalgae (*Enteromorpha flexuosa*) were collected from the coastal region of the Red Sea, on shores between Quoseir and Marsa-Alam, Egypt.

### 2.2. Methods

#### 2.2.1. Collection and Preparation of Macroalgae Samples

The collected samples were immediately taken to the laboratory in a polythene container with natural seawater to avoid evaporation. *Galaxaura elongata*, *Turbinaria ornata*, and *Enteromorpha flexuosa* were primarily washed with tap water to remove the trash, salts, and sticking epiphytes. Then they were washed several times in sterile distilled water, dried for several days in the shade at room temperature, ground either mechanically or by electric mixer until they became a powder ready for various extraction methods, and stored in a dark place away from moisture. Herbarium of the algal species was identified and saved.

#### 2.2.2. Preparation of Aqueous Seaweed Extract

##### Preparation of *Galaxaura elongata* Extract

Two grams of dried *Galaxaura elongata* seaweed powder were added to 100 mL of ethanol, mixed well, and filtered with Whatman No.1 filter paper to squeeze the *Galaxaura elongate*. The filtrate was applied to synthesize biogenic Ag-NPs.

##### Preparation of *Turbinaria ornata* Extract

Ten grams of *Turbinaria ornata* powder was added to 100 mL of deionized water; the fresh algal extract of *Turbinaria ornata* was prepared in boiling water for 15 min. After that, the extract was cooled and filtered through cotton tissue, then filtered through Whatman No.1 filter paper and held at 4 °C for Ag-NPs biosynthesis.

##### Preparation of *Enteromorpha flexuosa* Extract

*Enteromorpha flexuosa* extract was produced by adding 5 g of *Enteromorpha flexuosa* algal powder to 100 mL of deionized water and boiling the mixture for 15 min. Then, the extract was cooled and filtered with a cotton tissue and Whatman No.1 filter paper. The filtrate was kept in a refrigerator for the biogeneration of Ag-NPs.

#### 2.2.3. Biosynthesis of Ag-NPs

##### Biosynthesis of Ag-NPs by *Galaxaura elongata*

Biosynthesis of Ag-NPs requires the addition of 100 mL pure *Galaxaura elongata* extract to 900 mL of [10^−3^ M] AgNO_3_ solution. By visual observing the color change from light yellow to brown, the bioreduction of silver nitrate into Ag-NPs was confirmed. Then, the formulated Ag-NPs solution was maintained at room temperature in dark conditions and the resulting colloidal solution of Ag-NPs was analyzed using the UV-Vis spectrophotometer.

##### Biosynthesis of Ag-NPs by *Turbinaria ornata*

For the biosynthesis of Ag-NPs, 100 mL of *Turbinaria ornata* was added to 900 mL of [10^−3^ M] AgNO_3_ solution and blended well for Ag^+^ ion reduction. The mixture was kept in room conditions until the color transformed from yellow to dark brown, considering the synthesis of Ag-NPs as evidence. The reduction of pure silver ions was controlled by measuring the solution’s UV-Vis spectrum at regular times after 1.0:10.0 sample dilution.

##### Biosynthesis of Ag-NPs by *Enteromorpha flexuosa*

For biosynthesis of Ag-NPs, 880 mL of [10^−3^ M] AgNO_3_ solution was mixed with 120 mL of *Enteromorpha flexuosa* extract in an Erlenmeyer flask. After Ag-NPs’ formation, the solution was incubated at room temperature in the dark.

#### 2.2.4. Purification and Drying the Biosynthesized Ag-NPs

The obtained Ag-NPs solution was sanitized by repeated centrifugation for 30 min at 6000 rpm, subsequently redistributing the pellet in deionized water. Later, Ag-NPs were dried and preserved.

#### 2.2.5. Characterization of Biosynthesized Ag-NPs

The bioreduction of silver ions was observed by visual assessment and measuring the UV-Vis spectra of the reaction medium within a wavelength of 250–1000 nm and was conducted using the LAMBDA 950 PerkinElmer UV/Vis/IR spectrophotometer (PerkinElmer Inc., Waltham, MA, USA). Zeta potentials of the prepared Ag-NPs were analyzed using Dynamic Light Scattering (DLS) (Zetasizer Nano ZN, Malvern Panalytical Ltd., (Malvern, WR14 1XZ, Malvern, UK) at a fixed angle of 173° at 25 °C. Using an EDX-equipped JSM-6510 (JEOL, Tokyo, Japan), energy dispersive X-ray (EDX) and a scanning electron microscope (SEM), morphological analyses were performed. To determine the crystalline structure and desired orientation of the prepared Ag-NPs, X-ray diffraction (XRD) measurements were captured by the EMPYREAN X-ray diffractometer with a wavelength (λ) of 0.15418 nm. The XRD patterns were documented at a scan speed of 2°/min by drop coating film on the glass substrate in a wide range of Bragg angles (30–80°).

#### 2.2.6. Catalytic Experiments

The photocatalytic action of biosynthesized Ag-NPs was initially investigated for its ability to decay dangerous dyes such as methylene blue, Congo red, safranin O, and crystal violet under sunlight irradiation at 10 ppm concentrations. The photocatalytic activities were measured using UV-Vis spectrophotometers at 664, 504, 520, and 570 nm, respectively. The photocatalytic effects of different doses of Ag-NPs [0.1, 0.25, 0.5, 0.75, and 1.0 mg/50 mL] in distilled water were measured at varying concentrations of methylene blue [5, 10, 15, 20, and 25 ppm]. The control sample was preserved without the addition of Ag-NPs in the same conditions.

The moderate concentration of the dye was typically a 10 ppm solution, and the most effective dose of NPs was 1.0 mg/50 mL; afterwards, the dispersion solution was placed under sunlight and tracked at various pH values and temperatures from 0 min to 240 min. The absorbance spectrum of the supernatant was consequently determined using the Perkin–Elmer spectrophotometer. The dye removal efficiency was calculated by Equation (1):

(1) $$\mathrm{Dye}\;\mathrm{removal}\;\%=\frac{A_{0}-A_{s}}{A_{0}}\times 100$$

where A_0_ is the absorbance of blank dye and A_s_ is the absorbance of the dye after being treated with Ag-NPs.

The photocatalytic dye removal was studied as a function of contact time, catalyst dose, and dye concentration. Moreover, kinetics and mechanisms of degradation were investigated. Furthermore, catalyst stability was evaluated as a function of reusability runs. Note that data are measured in triplicates, and average results with a standard deviation of less than 5% are reported.

#### 2.2.7. Statistical Analysis

The photocatalytic dye removal data, which determine the ideal contact time, light impacts, catalyst dose, and dye concentration, were expressed as mean ± standard deviation of three replications. One-way analysis of variance (ANOVA) was conducted and was followed by Tukey’s test α = 0.05. This action was carried out by the statistical program for social sciences (SPSS) v. 20 programs.

## 3. Results and Discussion

### 3.1. Samples Characterization

**Figure 1:**
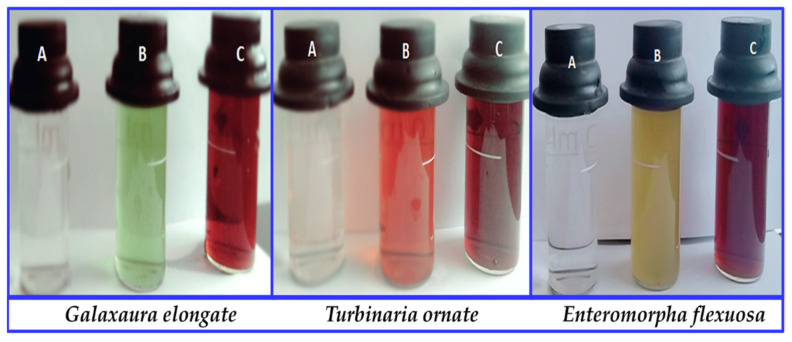
Visual observation of macroalgae based Ag-NPs synthesis (**A**) 10^−3^ M of AgNO_3_ solutions; (**B**) macroalgae extract of *Galaxaura elongata*, *Turbinaria ornata*, and *Enteromorpha flexuosa*, respectively, to AgNO_3_ solution; (**C**) formation of Ag[GE]-NPs, Ag[TE]-NPs, Ag[EE]-NPs, respectively.

To investigate their chemical and physical properties, the synthesis of Ag-NPs from *Galaxaura elongata*, *Turbinaria ornata*, and *Enteromorpha flexuosa* was verified and characterized using UV-Vis, SEM, EDX, and XRD. The observation of brown color is the first indication of the excitation of surface plasmon vibrations which is missing in the bulk material of [10^−3^ M] AgNO_3_, whereas color preservation was detected in the truancy of algal extract as shown in Figure 1. This means that marine red macroalgae (*Galaxaura elongata*), marine brown macroalgae (*Turbinaria ornata*), and marine green macroalgae (*Enteromorpha flexuosa*) extracts are the reducing agents.

#### 3.1.1. Optical Properties

**Figure 2:**
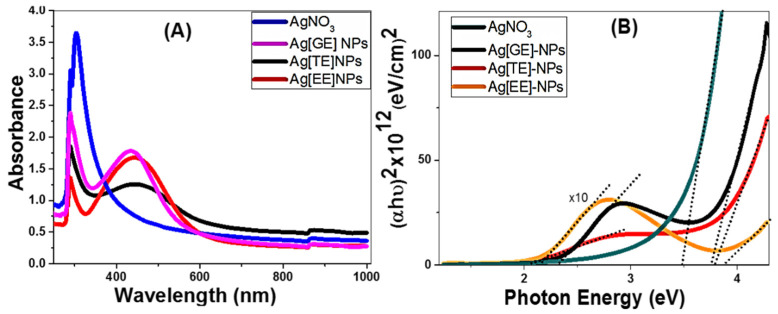
(**A**) UV-Vis spectra of different forms of macroalgae biosynthesized Ag-NPs and (**B**) the bandgap samples.

The UV-Vis absorbance spectra were measured and presented in Figure 2 to identify the surface plasmon resonance in the range of 250–1000 nm. Surface plasmon resonance (SPR) is a cooperative excitation of the electrons in the conduction band around the surface of the nanoparticles. AgNO_3_ showed two convoluted absorption bands in the UV region at 288 nm and 304 nm. The peak at 288 nm becomes weaker after the use of macroalgae for the processing of Ag[GE]-NPs, Ag[TE]-NPs, and Ag[EE]-NPs, and its FWHM is strongly reduced. For Ag[GE]-NPs, Ag[TE]-NPs, and Ag[EE]-NPs, a strong and wide absorption band centered at ~444 nm was observed to extend between 328 nm and 580 nm. This band corresponds to the absorption by colloidal Ag-NPs due to the excitation of surface plasmon oscillations for nanoparticles with sizes less than 100 nm [32,33]. The Ag[EE]-NPs and Ag[GE]-NPs showed stronger SPRs than Ag[TE]-NPs.

In order to predict the photophysical and photochemical properties of the nanocatalysts, a precise estimation of the bandgap energy is critical. The lowest energy required to excite an electron from the valence band to the conduction band is itemized by the bandgap energy of macroalgae-based Ag-NPs. The bandgap energy, Eg, of macroalgae-based Ag-NPs is directly calculated from the UV-visible absorption spectra using the Tauc Equation (2) [34,35]:

(2) $$\alpha =\frac{{\left(h\nu -\mathrm{Eg}\right)}^{\;1/2}}{h\nu }$$

where hν is incident photon energy and α is the absorption coefficient, which is calculated from Equation (3):

(3) α=2.303 × 103Aβ/1C

where β is the density of Ag[GE]-NPs, Ag[TE]-NPs, and Ag[EE]-NPs, l is the path of the quartz cell (1.0 cm), and C is the concentration.

The energy gap values were determined by extending the linear portions of the plots in Figure 2B to intersect the *X*-axis. The obtained values of Eg are 3.75 and 2.3 eV for Ag[GE], 3.78 and 2.04 eV for Ag[TE], and 3.87 and 2.15 eV for Ag[EE], versus only one value detected for AgNO_3_ at 3.47 eV. In addition to the presence of a wide bandgap, the engineered bandgap within the visible light range (<2.2 eV) for TE and EE bio-capped Ag-NPs makes them more suitable for solar energy applications.

#### 3.1.2. Mechanism of Silver Nanoparticle Biosynthesis

The Ag-NPs were originated from AgNO_3_ by the bottom-up method wherein atoms or particles were collected for molecular nanometer forms. With the addition of Egyptian marine macroalgae crude extract to AgNO_3_, the color altered from colorless to greenish, light yellow, whitish brown, red, and finally dark brown, which certifies the reduction of Ag^+^ to Ag^0^ using the biomolecules of *Galaxaura elongata*, *Turbinaria ornata*, and *Enteromorpha flexuosa* extracts. This marine macroalgae contains alkaloids, flavonoids, phenolic compounds, proteins, and sugars. Phenolic combinations and flavonoids are efficient reducing agents where proteins and particular other phytochemicals are capping agents for Ag-NPs [36]. The enol in flavonoid and phenolic compounds may release electrons by breaking the O–H bond, and this free electron may be used to reduce Ag^+^ to Ag^0^. The protein molecule in the extract is also thought to function as a capping and stabilizing agent [37].

#### 3.1.3. Energy-Dispersive X-ray Spectroscopy and Zeta Potential Analyses

**Figure 3:**
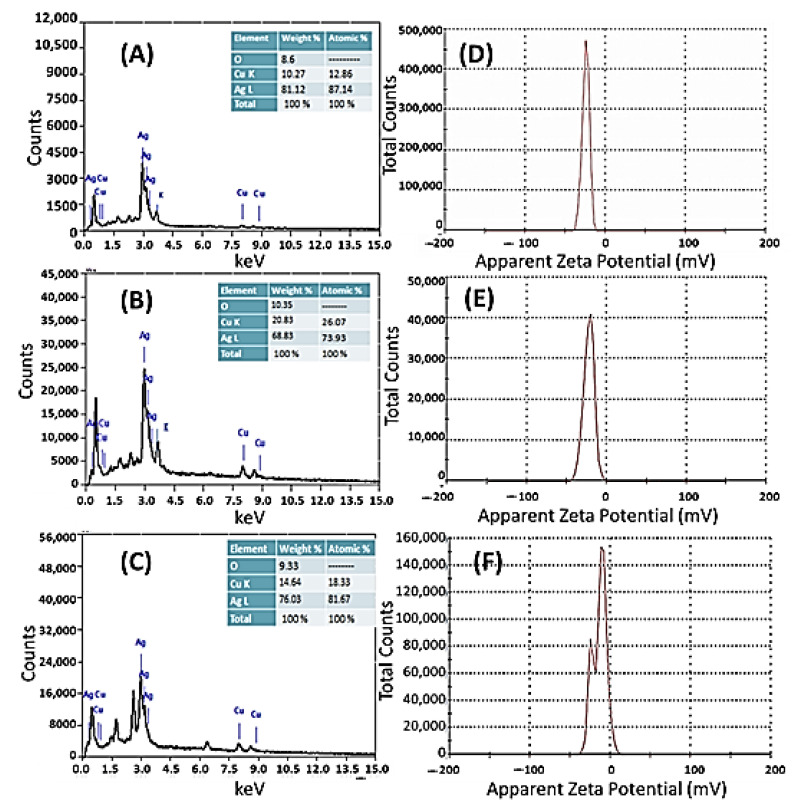
EDX spectra of biosynthesized (**A**) Ag[GE]-NPs, (**B**) Ag[TE]-NPs, and (**C**) Ag[EE]-NPs; and zeta potential distributions of (**D**) Ag[GE]-NPs, (**E**) Ag[TE]-NPs, and (**F**) Ag[EE]-NPs.

The EDX spectra (Figure 3) of the biosynthesized Ag[GE]-NPs, Ag[TE]-NPs, and Ag[EE]-NPs indicate the presence of a Ag signal as the major ingredient element. Additional signals are detected, such as O, Cu, Si, K, and other trace elements, thus implying the presence of the residual phytoconstituents of *Galaxaura elongata* as a capping ligand on the surfaces of the NPs (Figure 3A). The Ag[TE]-NPs and Ag[EE]-NPs EDX have a parallel component, as the basic reducing agents are marine macroalgae *Turbinaria ornata* and *Enteromorpha flexuosa* (Figure 3B,C) with O, Cu, and Si elements for Ag[EE]-NPs. The O signal results are from 0.5 Kev, minor signal 2.0 Kev for Si, major signals 2.8, 3.0 Kev for Ag, and 8.0, 8.4 for Cu. The inset tables of Figure 3 show the relative ratios of Ag, Cu, and O signals. The highest ratio of Ag (81.12%) is obtained for Ag[GE]-NPs, which indicates the high purity of *Galaxaura elongata* as a reducing and capping agent. In general, the detection of minor oxygen, copper, and other elements, based on the EDX study and relative to the highest Ag signal, confirms the presence of *Galaxaura elongata*, *Turbinaria ornata*, and *Enteromorpha flexuosa* biomolecules on the surface of the synthesized Ag-NPs [38,39,40].

The electrical potential between the inner Helmholtz layer at the particle’s surface and the bulk liquid in which the particle is suspended is known as the zeta potential. Figure 3D–F shows the zeta potential for biogenic Ag[GE]-NPs (−24.1 mV), Ag[TE]-NPs (−22.0 mV), and Ag[EE]-NPs (−13.3 mV). The obtained data indicate that the NPs’ surface is negatively charged and dispersed in the medium. Moreover, the negative value verifies the repulsion among the particles and indicates that macroalgae-based Ag-NPs are extremely stable.

#### 3.1.4. Scanning Electron Microscope (SEM)

**Figure 4:**
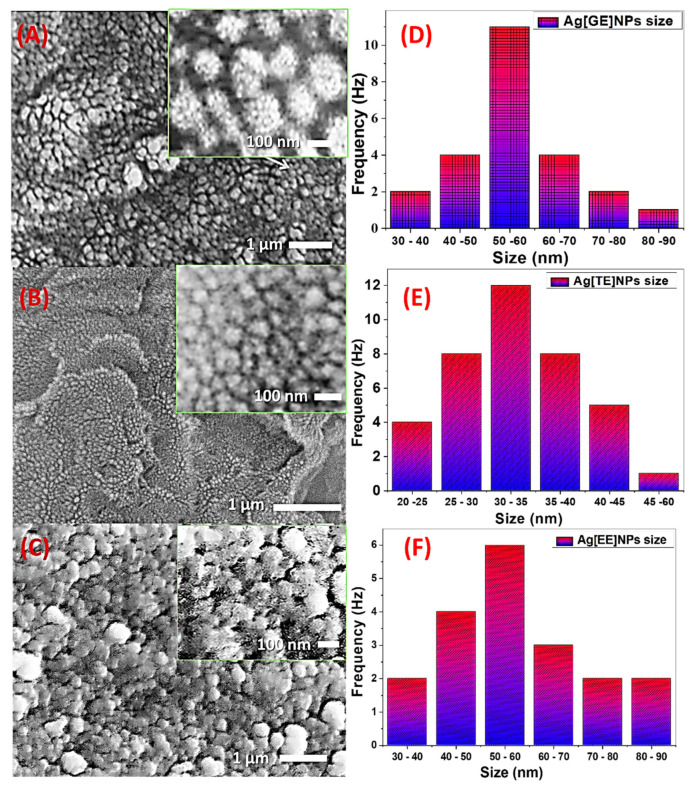
SEM images and particle size distribution of biosynthesized (**A**,**D**) Ag[GE]-NPs, (**B**,**E**) Ag[TE]-NPs, and (**C**,**F**) Ag[EE]-NPs.

Figure 4A–C displays the SEM images of Ag[GE]-NPs, Ag[TE]-NPs, and Ag[EE]-NPs. These images show the assembly of almost spherical Ag-NPs with nonuniform size. Figure 4A shows a high density of randomly distributed Ag[GE]-NPs and each one consists of agglomerated ultrafine spherical nanoparticles as shown in the inset. The particle size distribution of this sample is shown in Figure 4D. The particle size ranges from 30 to 90 nm with an average value of 55 nm. The agglomerates in the inset also showed nanoporous features resulting during the self-assembly of the fine nanoparticles.

Figure 4B displays an extraordinary density of disseminated Ag[TE]-NPs and each one is involved in agglomerated spherical nanoparticles. The A particle size distribution of this sample is shown in Figure 4E. The particle size of Ag[TE]-NPs ranges widely from 20 to 60 nm with an average value of 32.5 nm. The agglomerates in the inset also presented nanoporous features product in the self-assembly of the fine NPs. Analogously, Figure 4C presents a great density of dispersed Ag[EE]-NPs and each one is contained in agglomerated spherical nanoparticles. The dissemination of particle size is displayed in Figure 4F. The Ag[EE]-NPs particle size is varied from 30 to 90 nm with a mean value of 55 nm.

#### 3.1.5. X-ray Diffraction Analysis

**Figure 5:**
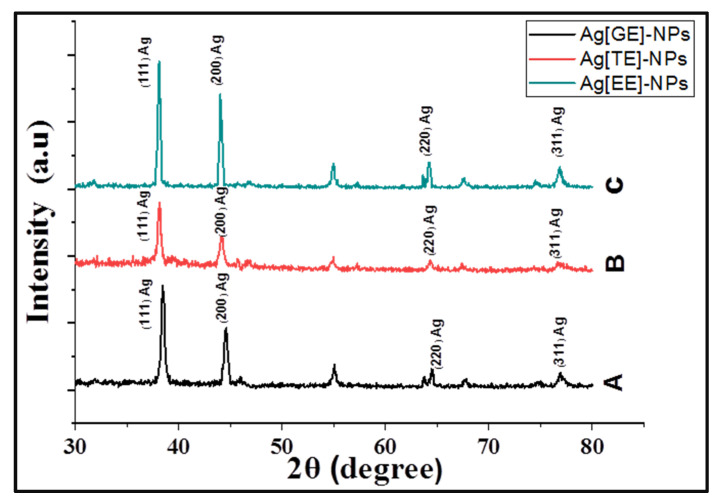
XRD patterns of macroalgae biosynthesized Ag-NPs: (**A**) Ag[ GE]-NPs, (**B**) Ag[TE]-NPs, and (**C**) Ag[EE]-NPs.

Crystallinity and different phases of biogenic Ag-NPs were identified and confirmed by X-ray diffraction analysis using the Empyrean system (Figure 5). The observed XRD peaks in a 2θ-range of 30–80°, shown in Table 1, correspond to the crystallographic planes of (111) Ag, (200) Ag, (220) Ag, and (311) Ag. Moreover, The XRD peaks at 2θ of 55.05° and 67.91° correspond to the crystallographic CuO planes of (020) and (022). The presence of these intense XRD peaks, especially along (111) and (200), confirm the crystalline nature of Ag[GE]-NPs, Ag[TE]-NPs, and Ag[EE]-NPs. The crystallinity of the biogenic Ag-NPs are in the following order: Ag[GE]-NPs > Ag[EE]-NPs > Ag[TE]-NPs.

The crystallographic crystallite size (Ds) and minimum dislocation density (DDm) are calculated by:

(4) $$\mathrm{Ds}\;\left(\mathrm{nm}\right)=\frac{0.9\times 0.154056\;\left(\mathrm{nm}\right)}{\mathrm{FWHM}\;\left(\mathrm{rad}\right)\;\times \;\cos\theta };\;\mathrm{DDm}\;\left(1/{\mathrm{nm}}^{2}\right)=1/{\left(\mathrm{Ds}\right)}^{2}];\;$$

where FWHM is the full width at half maximum [41]. The obtained data are reported in Table 1. The crystallite size for Ag using the preferred (111) orientation is 18.65, 18.47, and 26.67 nm for Ag[GE]-NPs, Ag[TE]-NPs, and Ag[EE]-NPs, respectively. The average crystallite size of Ag is 21.33, 22.33, and 25.61 nm for Ag[GE]-NPs, Ag[TE]-NPs, and Ag[EE]-NPs, respectively; the crystallite size for Cu signals is 35.95, 26.48, and 24.54 nm, respectively. From the reported DDm values in Table 1, the Ag[EE]-NPs showed the lowest dislocation densities for the professional crystallographic planes, (111) and (200), of Ag-NPs.

### 3.2. Dye Degradation

**Figure 6:**
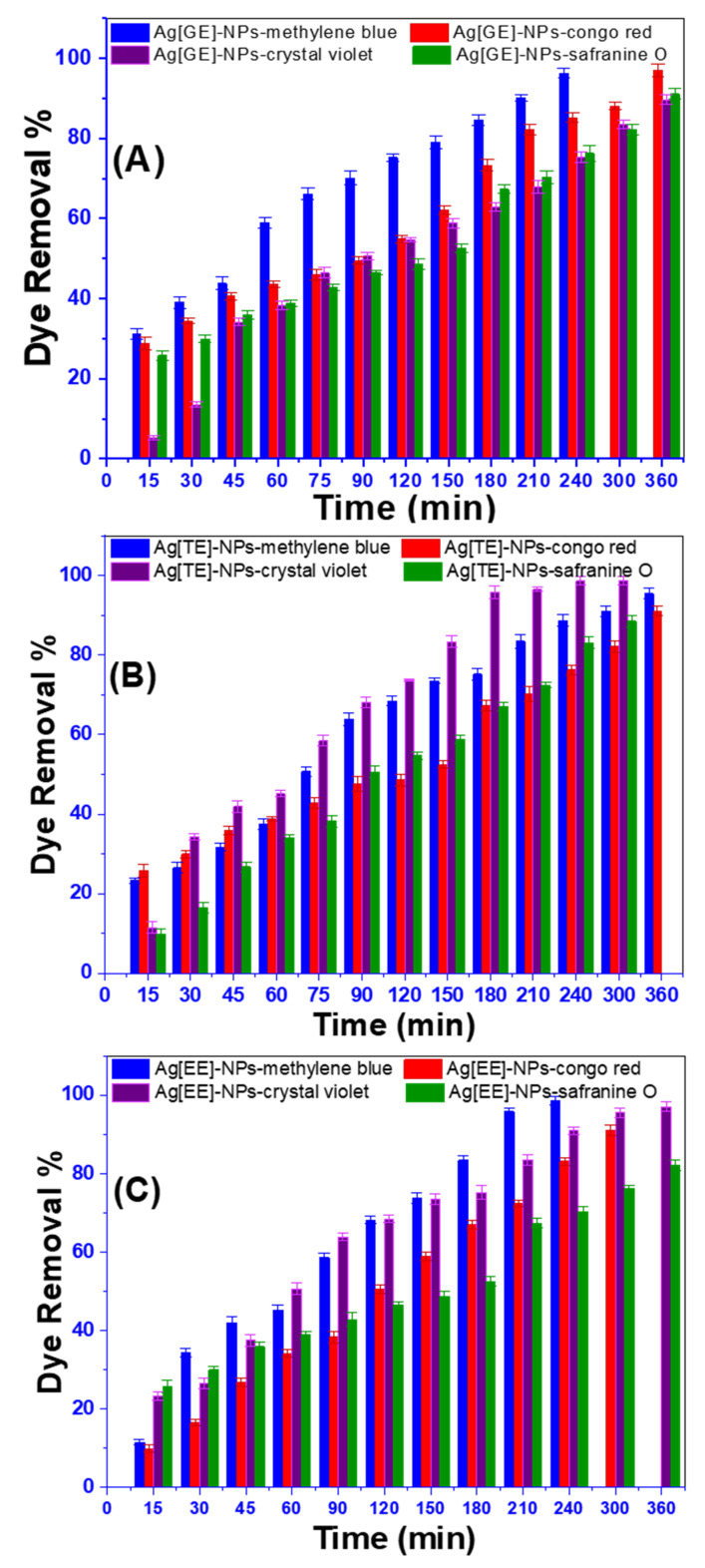
The degradation of 10 ppm methylene blue, Congo red, crystal violet, and safranin O using (**A**) Ag[GE]-NPs, (**B**) Ag[TE]-NPs, and (**C**) Ag[EE]-NPs. Values are mean ± SD, *n* = 3, using Tukey’s test *p* < 0.05.

Methylene blue is one of the most undesirable wastewater pollutants and poses a potential threat to the environment. It is widely utilized in textile industries for various objects [42]. Although Congo red is extremely lethal and devastating to plants and humans, it is one of the most used azo dyes in fabric manufacturing and is also used as an indicator in different industries. Safranin O is a category of heterocyclic azine dyes derived from phenazine, which may have toxic effects on the marine environment due to the presence of this dye in wastewater. Crystal violet dye is a triaryl methane dye used for various processes, such as textile dyeing, the coloring of paper, and other purposes. Therefore, the application of Ag[GE]-NPs, Ag[TE]-NPs, and Ag[EE]-NPs for the degradation of these four dyes is highly indispensable. In other words, the catalytic activities of biosynthesized Ag[GE], Ag[TE], and Ag[EE] NPs were explored by degrading 10 ppm methylene blue, Congo red, safranine O, and crystal violet aqueous solutions as shown in Figure 6. The catalytic degradation of these dyes was monitored using a UV-visible spectrophotometer by measuring the maximum absorbance of the investigated dyes at different time intervals.

From the displayed data in Figure 6, we can conclude that the use of Ag[EE]-NPs is a remarkable nanophotocatalyst and removes about 98.61 ± 1.06% of methylene blue from Ag[GE]-NPs with 97.03 ± 1.09% and Ag[TE]-NPs with 95.48 ± 1.37%. Moreover, Ag[TE]-NPs are the most effective catalysts in Congo red degradation with 98.76 ± 1.07% of Ag[GE]-NPs and Ag[EE]-NPs. However, in the case of crystal violet and safranine O, the results of Ag[GE]-NPs and Ag[TE]-NPs are similar, with a smaller removal ratio of Ag[EE]-NPs after 360 min. This is thanks to its high ability as a reduction and capping agent of *Galaxaura elongata*, *Turbinaria ornata*, and *Enteromorpha flexuosa* which have variable bio-ingredients able to initiate electron-hole pairs and initiate free hydroxyl radicals, such as •OH. Since the marine macroalgae-based Ag-NPs are incredibly effective in methylene blue dye degradation activity, this enhances the focus on different parameters that affect the rate of methylene blue dye degradations.

#### 3.2.1. Optimization of Variables in the Photocatalytic Degradation Process

In this work, we studied the impacts of various operational changes, such as effects of adsorption in the dark, photodegradation under sunlight, catalyst dose, dye concentration, temperature, pH, and stirring in the methylene blue dye removal process by using innovative macroalgae-based Ag-NPs photocatalysts.

#### 3.2.2. Impacts of Ag-NPs on Methylene Blue in Dark and Solar Light

**Figure 7:**
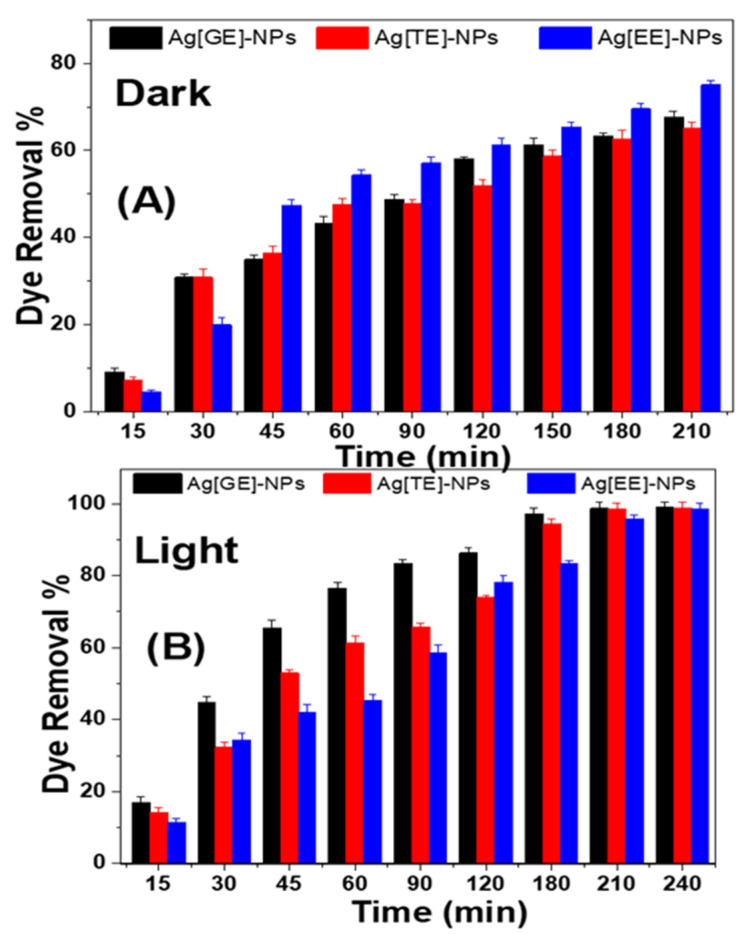
Catalytic activity of Ag[GE]-NPs, Ag[TE]-NPs, and Ag[EE]-NPs; (**A**) adsorption in the dark and (**B**) photodegradation under the sunlight of 10 ppm methylene blue dye solutions. Values are mean ± SD, *n* = 3, using Tukey’s test *p* < 0.05.

The catalytic activity of the biosynthesized Ag-NPs was evaluated by the degradation of methylene blue in the dark (adsorption) and in solar irradiation (photocatalytic). Methylene blue dye degradation was visually observed as a gradual disappearance of the dye color, turning from dark blue to colorless. In adsorption experiments (dark), Figure 7A reveals that the degradation process by Ag[GE]-NPs, Ag[TE]-NPs, and Ag[EE]-NPs is slow, while under solar irradiation, shown in Figure 7B, the photodegradation process was faster with the same time and other conditions. These results signify an evident augmentation in the photocatalytic properties of the macroalgae-based Ag-NPs’ capacity as compared to the adsorption capacity of methylene blue. The highest degradation ratio is 98.97 ± 1.03% for Ag[GE]-NPs, then 98.79 ± 1.20% for Ag[TE]-NPs, and finally 98.60 ± 1.30% for Ag[EE]-NPs. However, in dark conditions, the highest adsorption capacity is 75.10 ± 1.47% for Ag[EE]-NPs, then 67.36 ± 1.33% for Ag[GE]-NPs, and finally 65.03 ± 0.92% for Ag[TE]-NPs. This is due to their great ability as a reduction and capping agent of Ag[GE]-NPs, which has more bio-ingredients, and is therefore able to generate an electron-hole pair and trigger free radicals (hydroxyl radicals: •OH) that are capable of undergoing secondary reactions with Ag[EE]-NPs and Ag[TE]-NPs.

#### 3.2.3. Effect of Catalyst Dose

**Figure 8:**
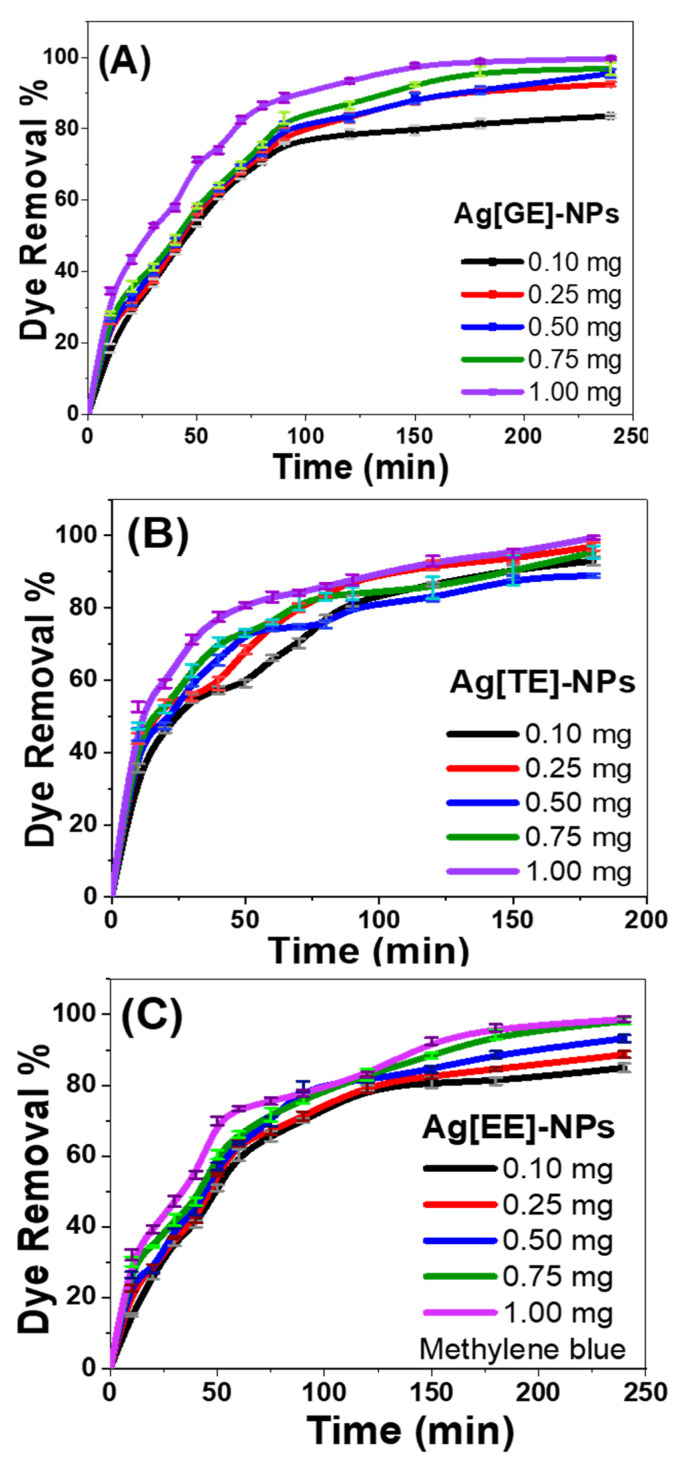
Photocatalytic activity of different doses of (**A**) Ag[GE]-NPs, (**B**) Ag[TE]-NPs, and (**C**) Ag[EE]-NPs on 10 ppm methylene blue dye removal. Values are mean ± SD, *n* = 3, using Tukey’s test *p* < 0.05.

The catalytic dose effects of Ag[GE]-NPs, Ag[TE]-NPs, or Ag[EE]-NPs on the removal of methylene blue dye were measured at various time intervals and are shown graphically in Figure 8A–C. The photocatalytic degradation of methylene blue dye indicates a superficial intensification in the removal ratio by raising the utilized dose [0.1, 0.25, 0.5, 0.75, and 1.0 mg] over all the studied periods from 0 to 240 min.

For Ag[GE]-NPs, shown in Figure 8A, the removal percentage augmented from 83.6% to ~100%, raising the utilized dose from 0.1 mg to 1.0 mg per 50 mL of methylene blue dye solution. A similar occurrence was observed for the photocatalytic degradation of methylene blue dye with Ag[TE]-NPs (Figure 8B), and Ag[EE]-NPs (Figure 8C). The high production of hydroxyl radicals and electron/hole pairs, along with the increase in the adsorption ability of the catalyst with increasing surface area and the obtainability of other active adsorption sites, may be correlated with improving the removal percentage by adding the affected dose [42,43,44]. With an increase in irradiation time, there is a commensurate rise in the quantity of methylene blue dye adsorbed, as well as an associated increase in the number of exciting electrons [42,43].

#### 3.2.4. Effects of Initial Methylene Blue Dye Concentrations

**Figure 9:**
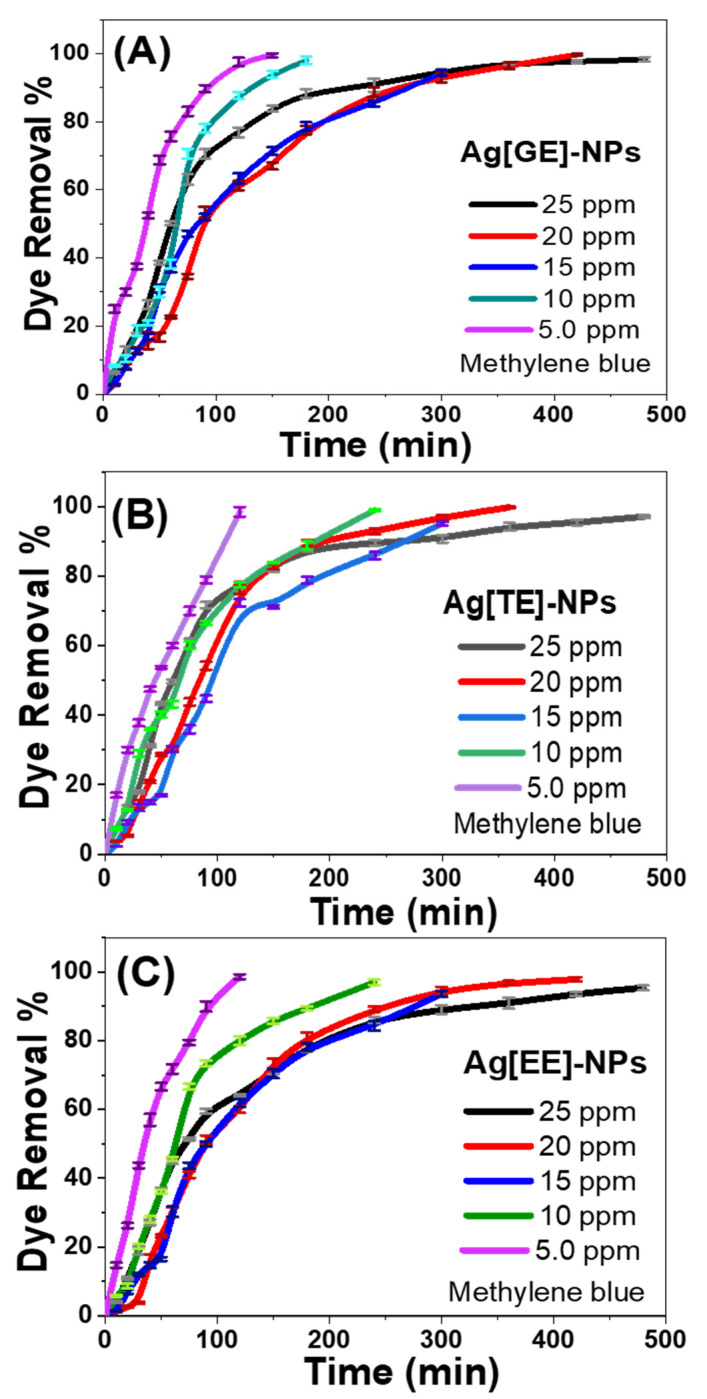
Photocatalytic activity of (**A**) Ag[GE]-NPs, (**B**) Ag[TE]-NPs, and (**C**) Ag[EE]-NPs at different initial methylene blue dye concentrations from 5 ppm to 25 ppm. Values are mean ± SD, *n* = 3, using Tukey’s test *p* < 0.05.

To scrutinize the initial methylene blue dye concentration effect on the photocatalytic process, the degradation procedure was performed in the presence of a definite dose of Ag nanocatalysts (20 mg/L) with variable initial concentrations of methylene blue in the series from 5 to 25 ppm; these data are depicted in Figure 9. The results assessment revealed that the photodegradation of methylene blue was appreciably determined by the initial dye concentration. As the increase in methylene blue concentration leads to a decrease in the density of the active sites on the nanoparticles’ surface, the total generated number of hydroxyl radicals declines, which may decrease the efficacy of photocatalytic activity. The elevation of the number of methylene blue dye particles likewise decreases the optical path length of the photons which reach the methylene blue stock solution.

For Ag[GE]-NPs (Figure 9A) the dye removal% is increased from 50.28 to 75.75% at 60 min and from 33.75 to 99.50% at 180 min by decreasing the starting dye concentration from 25 ppm to 5 ppm. For Ag[TE]-NPs (Figure 9B) the dye removal% is increased from 49.7 to 60% at 60 min and from 82.65 to 98.5% at 180 min by decreasing the initial dye concentration from 25 ppm to 5 ppm. For Ag[EE]-NPs (Figure 9C) the dye removal% is increased from 45.08 to 71.75% at 60 min and from 64.16 to 98.6% at 180 min by decreasing the initial dye concentration from 25 ppm to 5 ppm. It is also clear from the data that all bio-capped Ag-NPs have maximum degradation (~100%) with less time at low dye concentration (5 ppm), but the rise in dye concentration increases the time required to degrade dyes.

#### 3.2.5. Effect of Temperature on Photodegradation and Calculations of Activation Energy, Enthalpy, and Entropy

**Figure 10:**
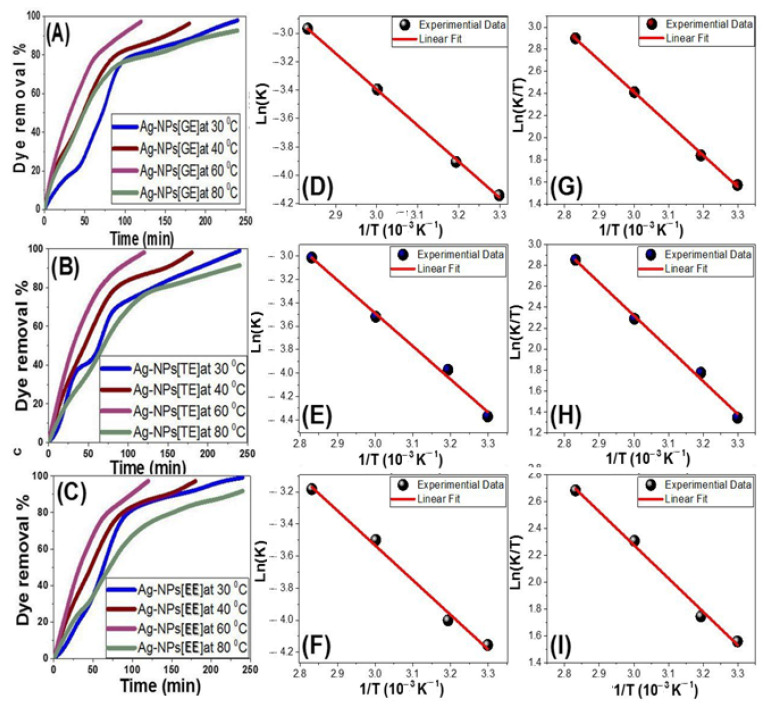
Photocatalytic activity at different temperatures and thermodynamic parameters calculations of methylene blue dye degradation; (**A**,**D**,**G**) Ag[GE]-NPs, (**B**,**E**,**H**) Ag[TE]-NPs and (**C**,**F**,**I**) Ag[EE]-NPs.

Temperature is an important factor that influences degradation capacities. To explore the temperature influence on photocatalytic progression, the photocatalytic performances of Ag[GE]-NPs, Ag[TE]-NPs, and Ag[EE]-NPs were examined at various temperatures ranging from 30 °C to 80 °C, as shown Figure 10. It was perceived that the percentage of dye removal increased along with raising the temperature from 30 °C to 60 °C, while the other conditions remained unchanged. At the same Ag-NPs concentration, the dye photo-degradation improved linearly with the increase in temperature from 30 °C to 60 °C.

For Ag[GE]-NPs, Figure 10A indicates that the dye removal percent is 97.97% at 30 °C after 240 min, whereas it became 97.22% at 60 °C after 120 min. For Ag[TE]-NPs, Figure 10B demonstrates that the dye removal% is 98.98% at 30 °C after 240 min and reached 98.14% at 60 °C after 120 min. For Ag[EE]-NPs, Figure 10C shows that the dye removal% is 99.08% at 30 °C after 240 min and is 97.22% at 60 °C after 120 min. According to Figure 10, the greatest temperature reached was 60 °C, whereas the maximum dye removal% is observed after a 2 h reaction time. The improvement in dye removal% with a temperature increase will elucidate the higher affinity of binding sites for dye particles at high temperatures. An increase in temperature causes dye molecules to move more and the delaying forces acting on them to decrease, therefore increasing the dye-binding capacity of the adsorbent. The observed trend of improved dye-removal capacity with increased temperature suggests that dye adsorption capacity via nanoparticles was kinetically controlled in an endothermic manner [45]. Molecules that are at a high temperature require additional thermal energy. While the increase in collision frequency is significant at higher temperatures, it only accounts for a small portion of the improvement in reaction rate. The proportion of reactant molecules with sufficient energy to react (energy greater than activation energy: E > Ea) is significantly higher. In addition to increasing the activation energy, as described above, rising temperatures cause improvements in the dye molecules’ kinetic energy that adjusts the collisions together with the catalyst surface. If the catalyst surface is vigorous, several free radicals will be present in the thin film surrounding the catalyst nanoparticles, causing fast, brilliant dye molecule reactions to arise; then, the molecules will run with no degradation.

The activation energy (Ea) was calculated from the Arrhenius plot of ln(k) against 1/T, while K was measured from the first-order plots, and T represents the Kelvin temperature as shown in Figure 10D–F. The temperature increase allows an efficient response to a more competitive e–/H+ recombination. The estimated values of Ea from Figure 10D–F are reported in Table 2. Figure 10D–F reveals that the Ea of Ag[GE]-NPs, Ag[TE]-NPs, and Ag[EE]-NPs are 21.10, 23.42, and 17.96 kJ/mol. In other words, the lowest Ea was obtained for Ag[EE]-NPs, and the highest value was for Ag[TE]-NPs. The positive activation energy means that the reaction is spontaneous and less energy intensive. This may be because the activated state is a well-solved structure formed between dye molecules and hydroxyl radicals that are reaction intermediates, which is also supported by positive activation entropy [46]. Thus, the related enthalpy (∆H*) and entropy (∆S*) are determined from Equations (5) and (6) [47,48].

(5) $$k=\frac{{\mathrm{TK}}_{B}}{h}\cdot {\;e}^{\frac{{\Delta S}^{*}}{R}}\cdot e^{\frac{-{\Delta H}^{*}}{\mathrm{TR}}}$$

ln (k/T) = ln(KB/h) + ΔS*/R − (ΔH*/R) (1/T)(6)

From the slope of the linear fitting of the relation between ln (k/T) and 1/T (Figure 10G–I), The obtained values of ΔH* and ΔS*are outlined in Table 2. The value of ΔH* is found to be −20.68 ± 1.11 kJ·mol^−1^ and from the intercept, the value of Δs* is found to be 213.78 ± 3.42 J·mol^−1^·K^−1^ for Ag[EE]-NPs, which are the lowest obtained values of ΔH and ΔS.

#### 3.2.6. Effect of Stirring

**Figure 11:**
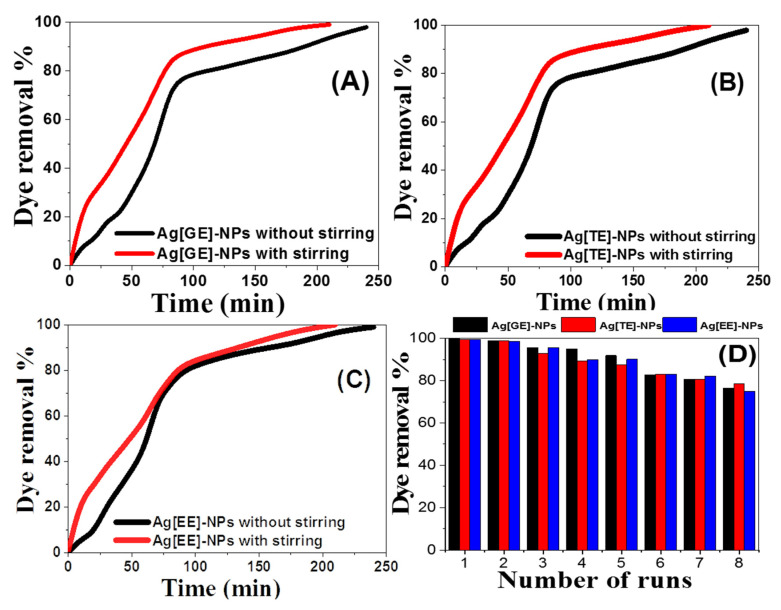
Effects of (**A**–**C**) stirring and (**D**) 8 runs of reusability on the photodegradation of 10 ppm methylene blue dye by 20 mg/L of the catalyst.

To scrutinize the effect of stirring on the methylene blue photocatalytic degradation rate via Ag-NPs, the investigation was inspected under optimal conditions: dye concentration of 10 ppm, 20 mg/L of Ag-NPs, and 30 °C. The findings in Figure 11A–C show that stirring increases the degradation proportion of methylene blue dye using the catalysts Ag[GE]-NPs, Ag[TE]-NPs, and Ag[EE]-NPs. At 200 rpm, the removal% after 180 min was increased to 97.65%, 95.3125%, and 96.09% for Ag[GE]-NPs, Ag[TE]-NPs, and Ag[EE]-NPs, respectively. This increase may be attributed to the increase in stirring intensity, which initially increases the overall dissolved oxygen between the layers of the solution. As a result, the increase in stirring intensity initially increases the overall dissolved oxygen between the layers of the solution. Therefore, the addition of dissolved oxygen has a critical impact on the formation of hydroxyl radicals. Stirring the solution to minimize the balance time increases the dispersion level of methylene blue particles to the nanocatalyst’s surface [48].

#### 3.2.7. Reusability of Ag-NPs for Methylene Blue Dye Degradation

The most serious aspect of the efficient application of any catalyst is its stability and reusability. Our nanocatalyst reusability consisted of more than eight runs, and it was investigated by mixing 20 mg/L of nanocatalyst with 10 ppm of methylene blue dye, shown in Figure 11D. The nanocatalyst powder was cleaned with distilled water after each run and dried at 60 °C for 60 min before reapplication. This was repeated for eight runs under sunlight within a time span from 15 to 240 min. The reusing efficiencies of Ag-NPs signify that after the eighth run, the removal percent was 76.36%, 78.58%, and 74.88% for the reusing process of biosynthesized Ag[GE]-NPs, Ag[TE]-NPs, and Ag[EE]-NPs, respectively, as illustrated in Figure 11D.

#### 3.2.8. The Effect of pH Value on Photocatalytic Activities of Biosynthesized Ag-NPs

**Figure 12:**
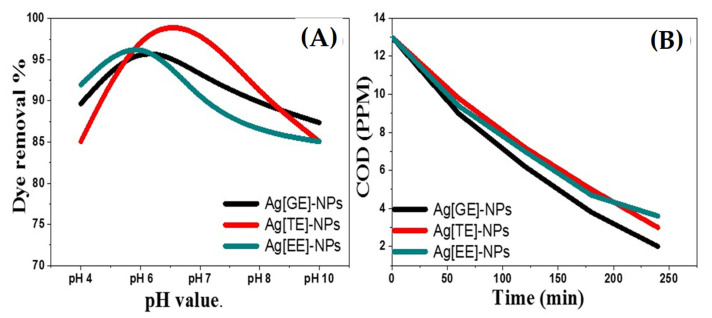
(**A**) The photocatalytic activity of 20 mg/L of Ag[GE]-NPs, Ag[TE]-NPs, and Ag[EE]-NPs as a function of the pH value of 10 ppm methylene blue solution; and (**B**) COD for biodegraded 10 ppm methylene blue by 20 mg/L of biosynthesized Ag[GE]-NPs, Ag[TE]-NPs and Ag[EE]-NPs.

The initial solution pH value is a key factor that affects the degradation of dyes because pH influences the surface charge properties of the catalyst [49]. The degradation of methylene blue dye was scrutinized at different pH values from 4 to 10 (Figure 12A). When the pH is higher than eight, the Ag-NPs become negatively charged according to Equation (7):

AgOH + OH− ➔ H_2_O + Ag^−^ (7)

At lower pH values, the number of positively charged adsorbent sites on the biogenic Ag-NPs’ surface increased according to Equation (8):

AgOH + H+ ➔ H_2_O + Ag^+^ (8)

Consequently, the dye removal process accelerated in the pH range from 6 to 7 significantly, and the electrostatic interaction between the negatively charged dye molecules and the binding sites on the surface of augmented Ag-NPs preferred the dye anions uptake. Analogously, at higher pH values, the Ag-NPs’ surface became more negatively charged, and hence the electrostatic repulsion between the dye molecule and the Ag-NPs’ surface sites increased, which led to a decrease in the uptake of dye anions.

As illustrated in Figure 12A, the photodegradation process was efficient at a particular pH value because of the change in the oxidation potentials; the maximum degradation ratios were 97.7, 98.85, and 99.54 respectively for Ag[GE]-NPs, Ag[TE]-NPs, and Ag[EE]-NPs at pH 6 but it decreased gradually in the alkaline medium until it became 87.3, 85.06, 85.05 at pH 10.

#### 3.2.9. Chemical Oxygen Demand (COD) of Biodegraded Methylene Blue Dye by Ag-NPs

The COD content in dye-polluted water is very high. The COD investigation shows only the complete waste load in the discharge of textile waste and the COD ratio supports evidence of the biodegradability of organic materials in wastewater samples [50]. Figure 12B shows the data describing the impact of Ag-NPs on COD biodegradability of methylene blue dye. The findings indicate that the COD decreases were 84.6%, 76.9% and 72.23% after the photocatalytic treatment of the industrial wastewater samples by Ag-NPs, which suggests a progressive change in the biodegradation process.

The COD degradation proficiencies of macroalgae-based Ag-NPs indicate that the elimination percentage of Ag[GE]-NPs was 84.6% because of the highly biogenic ingredient of *Galaxaura elongata*, then *Turbinaria ornata* and *Enteromorpha flexuosa*, as illustrated in Figure 12B.

#### 3.2.10. The Kinetic Reactions

The kinetic models which include first and second order were performed to explain the removal action of methylene blue using different forms of macroalgae biosynthesized Ag-NPs. Calculations of the computed kinetic models were stated as in Equations (9) and (10) for the first-order and second-order kinetic models individually [51].

(9) $$\frac{\mathrm{dc}\;}{\mathrm{dt}}=-K_{1}\;C$$

(10) $$\frac{\mathrm{dc}}{\mathrm{dt}}=-K_{2}C_{2}$$

where the methylene blue dye concentration is C and t is the reaction time. The kinetic rate constants are K_1_ for the first-order reaction kinetics and K_2_ for the second-order reaction kinetics. With the addition of Equations (9) and (10), the linear appearance of the kinetic reactions for the first-order and second-order kinetic patterns can be contributed [51].

**Figure 13:**
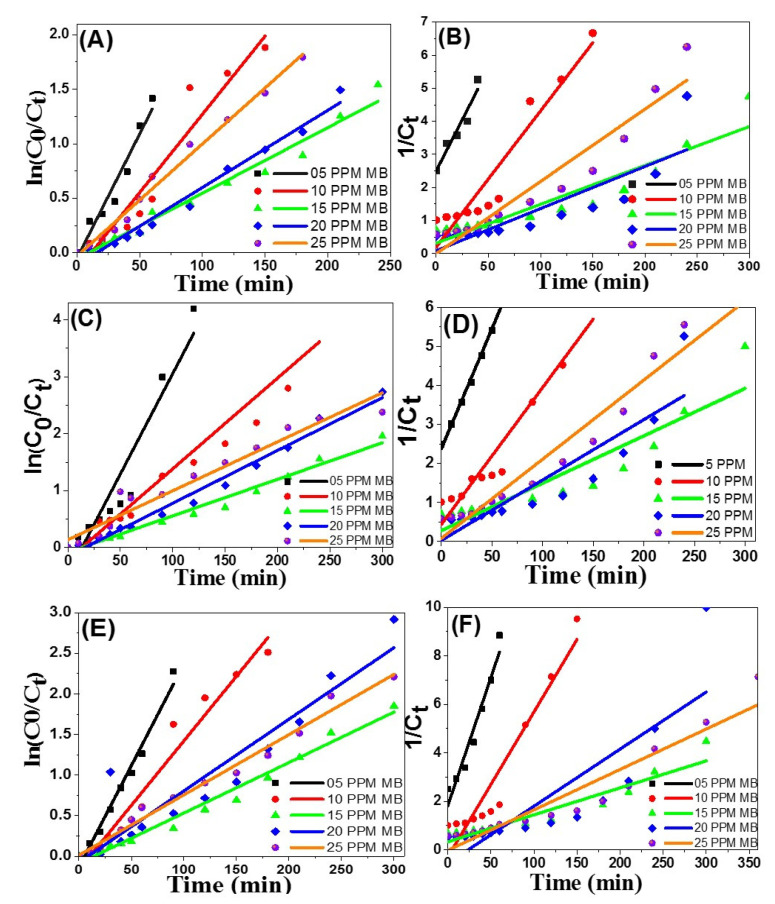
First- and second-order kinetic plots; (**A**,**B**) for Ag[GE]-NPs, (**C**,**D**) for Ag[TE]-NPs and (**E**,**F**) for Ag[EE]-NPs in the photo-degradation of methylene blue dye.

The first-order kinetic pattern was obtained from the linear relation between ln(C_0_/C_t_) and time (t) as illustrated in Figure 13A,C,E. The second-order kinetic model was analyzed from the linear relation between 1/C_t_ and time (t) as demonstrated in Figure 13B,D,F. The kinetic factors for the chosen models were scheduled in Table 3. The correlation coefficient (R_2_) is an assessment that governs the point at which two variables are linked to each other. Through R_2_ evaluation for the two models, it can be decided that the methylene blue decaying within Ag[GE]-NPs, Ag[TE]-NPs, or Ag[EE]-NPs more closely fit with the second-order kinetic model than with the first-order kinetic model for all examined methylene blue dye concentration [5–25 mg/L].

Declining the decaying percentage by increasing the original dye concentration may be allied to the elevated quantity of adsorbed methylene blue dye on the nanocatalyst surface beyond the precarious limit. Hence, obtainable active sites on the nanocatalysts’ surface will be lowered, and in turn, the created hydroxyl radicals will be reduced [52]. Furthermore, increasing the primary dye concentration acts as a blocking barrier between the incident photons and the surface of the nanocatalyst. As a result of the decreased quantity of absorbed photons using Ag-NPs, the degradation level will decrease. The kinetic findings of methylene blue degradation consuming Ag[GE]-NPs display results similar to the results obtained for degradation using Ag[TE]-NPs or Ag[EE]-NPs as illustrated in Table 3 and depicted in Figure 13. The calculated K_1_ values of photocatalytic degradation using Ag[TE]-NPs are higher than those for the degradation using Ag[GE]-NPs or Ag[EE]-NPs, but the rate constant K_2_ for the photocatalytic degradation using Ag[EE]-NPs are higher than those for the degradation using Ag[GE]-NPs or Ag[EE]-NPs. 

#### 3.2.11. Degradation Mechanism and Effect of Scavengers on the Photocatalytic Mechanism

Photocatalysis is the demonstration of a catalyst that involves photoreaction acceleration. The mechanism of the oxidizing species formed during methylene blue photodegradation has been studied extensively; photocatalytic activity is dependent on the catalyst’s ability to generate electron-hole pairs. The photocatalytic degradation process consists of three steps: (a) dye adsorption, (b) light absorption utilizing the catalyst, and (c) charge transference reactions that generate the radicals essential for dye breakdown [53].

Because dye molecules are electrophilic, their reduction capacity increases when they are adsorbed by nanoparticles. As a result, when species are adsorbed on nanoparticles, they become more -ve for extract molecules and more +ve for dye molecules. The transfer of electrons from the reducing agent to the dye particles occurs via metal nanoparticles, where excited surface electrons interact with dissolved oxygen particles to produce hydroxyl radicals while allowing Ag^+^ ions to cooperate with the anionic dye [54,55], resulting in the destruction of the dye chromosphere and minor species formation. As a result, macroalgae-based Ag-NPs appear to be a highly promising photocatalytic agent for dye degradation under sunlight.

**Figure 14:**
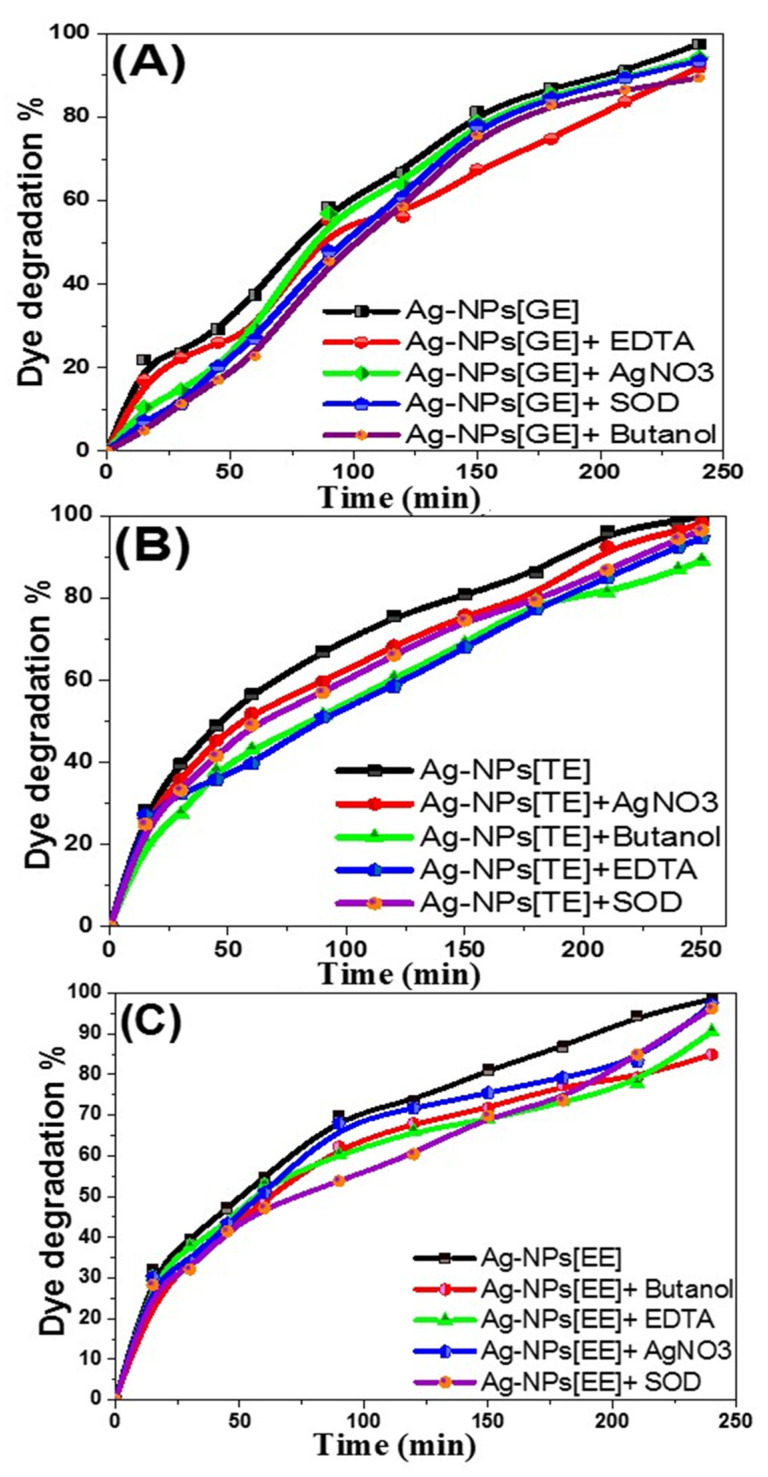
Trapping investigations of active species during the photocatalytic degradation of methylene blue dye in excess of different free radicals with biogenic Ag-NPs; (**A**) Ag[GE]-NPs, (**B**) Ag[TE]-NPs, and (**C**) Ag[EE]-NPs.

For further clarification on methylene blue degradation mechanisms using macroalgae-based Ag-NPs, the photo-generated electron-hole pairs in the photocatalytic progression are identified within trapping investigations of radicals and holes. This process is conducted by using 1.0 mmol of scavengers for the generated active radicals as ethylene diamine tetra-acetic acid sodium (EDTA-2Na), aliphatic alcohols, H_2_O_2,_ and AgNO_3_ scrutinize the production, and the roles of h^+^, •OH, e, and •O_2_^−^ blocked degrading methylene blue in an attempt to illuminate the reaction mechanism over the visible-light approach of different biogenic Ag-NPs. As showed in Figure 14A–C, paralleled with no scavenger at similar condition, the photocatalytic activity in the Ag-NPs is critically repressed by the addendum of butanol (•OH radical scavenger) and EDTA-2Na (hole scavenger), signifying the principal roles of •OH and h^+^ for methylene blue degradation. The adding of H_2_O_2_ (•O_2_^−^ radical scavenger) and superoxide dismutase (•O_2_^−^ scavenger) revealed lower influences in the methylene blue photo-degradation process. The result displays that the methylene blue degradation by biogenic macroalgae-based Ag-NPs has hindered the incidence of these scavengers with the subsequent effects of Butanol > EDTA-2Na > SOD > AgNO_3_ [43].

## 4. Conclusions

We can infer from this study that using extracts of *Galaxaura elongata*, *Turbinaria ornata*, and *Enteromorpha flexuosa* was an effectively novel and convenient way of biosynthesizing silver-based nanoparticles. These extract components were substantiated to be a strong reducing agent, as well as a capping agent for the biosynthesized Ag-NPs. The prepared Ag[GE]-NPs, Ag[TE]-NPs, and Ag[EE]-NPs were applied successfully for the photocatalytic degradation of four different hazardous dyes in an economic and eco-friendly manner. Also, due to their extremely high surface area, the degradation potential was improved to reach ~100%, and the migration rate of electrons/holes to the surface of the nanoparticles was accelerated. Degradation efficacy is enhanced with varying time intervals which can broadly be used in the treatment of industrial dye waste. The efficiency of photocatalytic dye removal was imitated using dye concentration, exposure time, catalyst dosage, and temperature. Moreover, the results reveal the high ability for reusing macroalgae-based Ag-NPs for more than 8 phytoconstituents runs in photocatalytic dye removal. The thermodynamic and kinetic parameters were also calculated. The dye degradation performed by the biogenic macroalgae-based Ag-NPs was hindered in the incidence of different free radical scavengers including the following order: Butanol > EDTA-2Na > SOD > AgNO_3_.

## Figures and Tables

**Table 1:** XRD crystallographic parameters of the macroalgae biosynthesized Ag-NPs.

Samples	Peaks		FWHM (Radian)	Ds (nm)	Average Ds (nm)	DDm (10^−3^ nm^−2^)
	2θ (Degree)	Planes				
**Ag[GE]-NPs**	38.472	(111) Ag	0.451	18.65	21.33	2.88
	44.558	(200) Ag	0.5418	15.84		3.99
	64.476	(220) Ag	0.289	32.48		0.95
	76.985	(311) Ag	0.5464	18.57		2.9
	55.046	(020) CuO	0.2130	42.04	35.95	0.57
	67.91	(022) CuO	0.32	29.92		1.12
**Ag[TE]-NPs**	38.111	(111) Ag	0.4506	18.47	22.33	2.93
	44.148	(200) Ag	0.541	15.84		3.99
	64.323	(220) Ag	0.2874	32.64		0.94
	76.817	(311) Ag	0.4534	22.35		2
	54.241	(020) CuO	0.379	23.54	26.48	1.8
	67.531	(022) CuO	0.468	20.41		2.4
**Ag[EE]-NPs**	38.095	(111) Ag	0.315	26.67	25.61	1.41
	44.059	(200) Ag	0.3337	25.67		1.52
	64.222	(220) Ag	0.2867	32.7		0.94
	76.846	(311) Ag	0.5482	17.41		3.3
	54.939	(020) CuO	0.3653	24.5	24.54	1.67
	67.566	(022) CuO	0.3886	24.58		1.66

**Table 2:** The thermodynamic parameters of the photocatalytic reaction of 20 mg/L of Ag[GE]-NPs, Ag[TE]-NPs, and Ag[EE]-NPs Ag-NPs for photodegradation of 10 ppm methylene blue dye.

Material	Ag[GE]-NPs	Ag[TE]-NPs	Ag[EE]-NPs
**The activation energy kJ/mol**	21.10 ± 0.39	23.42 ± 1.28	17.96 ± 1.11
**Enthalpy(∆H) kJ·mol^−1^**	−23.82 ± 0.412	−26.14 ± 1.27	−20.68 ± 1.11
**Entropy (∆S) J·mol^−1^·K^−1^**	224.36 ± 1.27	230.54 ± 3.91	213.78 ± 3.42

**Table 3:** Kinetic model parameters for the photodegradation of different initial concentrations of methylene blue dye by 20 mg/L of Ag[GE]-NPs, Ag[TE] ]-NPs, and Ag[EE] ]-NPs.

Material	Kinetic Models		5 mg/L	10 mg/L	15 mg/L	20 mg/L	25 mg/L
**Ag[GE]**	First-order kinetic mode	R^2^	0.9524	0.9325	0.97563	0.98041	0.98964
		K_1_ (min^−1^)	0.0228	0.01435	0.00603	0.00709	0.01028
		K_1_· R^2^	0.02171	0.01338	0.00588	0.00695	0.01017
	Second-order kinetic mode	R^2^	0.93117	0.91202	0.88416	0.73867	0.92093
		K_2_ (L/mol·min)	0.0619	0.04124	0.01177	0.0127	0.0218
		K_2_· R^2^	0.05767	0.03762	0.01041	0.0094	0.02014
**Ag[TE]**	First-order kinetic mode	R^2^	0.90855	0.92507	0.98099	0.98415	0.94586
		K_1_ (min^−1^)	0.0353	0.01593	0.00642	0.00927	0.0086
		K_1_· R^2^	0.03207	0.01474	0.0063	0.00912	0.00813
	Second-order kinetic mode	R^2^	0.99272	0.94107	0.86014	0.80818	0.96284
		K_2_ (L/mol·min)	0.06153	0.03506	0.01214	0.01547	0.02028
		K_1_· R^2^	0.06108	0.03299	0.01044	0.0125	0.01953
**Ag[EE]**	First-order kinetic mode	R^2^	0.98167	0.96358	0.98073	0.8855	0.98633
		K_1_ (min^−1^)	0.02503	0.01589	0.00622	0.00882	0.00742
		K_1_· R^2^	0.02457	0.01531	0.0061	0.00781	0.00732
	Second-order kinetic mode	R^2^	0.95132	0.91761	0.88492	0.72758	0.90685
		K_2_ (L/mol·min)	0.10569	0.05981	0.01112	0.02354	0.01667
		K_2_. R^2^	0.10055	0.05488	0.01713	0.00984	0.01512

## Data Availability

The data presented in this study are available on request from the corresponding author.
